# Contribution of the Proximal Nerve Stump in End-to-side Nerve Repair: In a Rat Model

**DOI:** 10.4055/cios.2009.1.2.90

**Published:** 2009-05-27

**Authors:** Jun Mo Jung, Moon Sang Chung, Min Bom Kim, Goo Hyun Baek

**Affiliations:** Department of Orthopedic Surgery, Yeson Hospital, Bucheon, Korea.; * Department of Orthopedic Surgery, Seoul National University College of Medicine, Seoul, Korea.

**Keywords:** Median nerve, Proximal stump, Peripheral nerve injury, End-to-side neurorrhaphy, Nerve recovery

## Abstract

**Background:**

The aim of this study was to evaluate the contribution of the proximal nerve stump, in end-to-side nerve repair, to functional recovery, by modifying the classic end-to-side neurorrhaphy and suturing the proximal nerve stump to a donor nerve in a rat model of a severed median nerve.

**Methods:**

Three experimental groups were studied: a modified end-to-side neurorrhaphy with suturing of the proximal nerve stump (double end-to-side neurorrhaphy, Group I), a classic end-to-side neurorrhaphy (Group II) and a control group without neurorrhaphy (Group III). Twenty weeks after surgery, grasping testing, muscle contractility testing, and histological studies were performed.

**Results:**

The grasping strength, muscle contraction force and nerve fiber count were significantly higher in group I than in group II, and there was no evidence of nerve recovery in group III.

**Conclusions:**

The contribution from the proximal nerve stump in double end-to-side nerve repair might improve axonal sprouting from the donor nerve and help achieve a better functional recovery in an end-to-side coaptation model.

End-to-end neurorrhaphy has had relatively satisfying results with regard to nerve regeneration since the introduction of microsurgery. The microsurgical end-to-end neurorrhaphy is currently regarded as the gold standard for nerve reconstruction.^1^,2) Even so, this procedure is not always applicable. In cases with old or extensive nerve injuries, nerve grafting or direct muscle neurotization has been used as an alternative procedure; however, these techniques do not produce as successful results as the end-to-end procedure. The end-to-side (ETS) neurorrhaphy^3^,4) was developed for these situations; it is a procedure where the distal stump of a transected nerve is sutured to an adjacent healthy nerve. The recovered axonal passage to the recipient nerve, through the coaptation site, has been demonstrated in animal experiments.^5^-8)

The hypothesis of our study was that the original neurons of severed nerves contribute to axonal regeneration after ETS neurorrhaphy. If this is the case, then a double ETS neurorrhaphy, which requires the additional coaptation of the proximal end of the severed nerve to the donor nerve, might be more effective in nerve repair than a single ETS neurorrhaphy. The grasping strength and the muscle contraction force of the finger flexor muscle of rats and the number of myelinated axons of the injured median nerve were measured to evaluate the contribution from the proximal nerve stump after ETS nerve repair on functional recovery.

## METHODS

### Materials

Healthy male mature Sprague-Dawley rats (average weight, 300 g) were used in the experiments. We used the median nerve as the recipient nerve and the ulnar nerve as the donor nerve. As the median nerve innervates solely the finger flexor muscle of the rat,9) this design facilitates functional assessment of the finger flexor muscle. In addition, as the ulnar nerve was kept intact, some sensation of the upper limb remained unaffected, and self mutilation or self-biting, due to the loss of sensation among the experimental animals, could be prevented.

### Methods

A total of 45 Sprague-Dawley rats were randomly divided into three groups, with 15 rats in each group. All surgery was performed under general anesthesia using sodium pentobarbital (50 mg/kg body weight) administrated intraperitoneally. The median and ulnar nerves were exposed using a 2 cm incision in the brachial area. A 1.5 cm segment of the right median nerve was removed at a point 1 cm proximal to the elbow joint. The distal stump of the median nerve was coapted to the ulnar nerve through an epineurial window (approximately 2 mm^2^) in an end-to-side fashion with 11-0 nylon sutures using an operating microscope (Zeiss S 22, OPMI 6-SFR, Zeiss, Munich, Germany). In group I, the proximal stump of the median nerve was coapted in an end-to-side fashion to the ulnar nerve, 2 cm proximal to the above mentioned coaptation site. In group II, the proximal stump end was pulled more proximally, ligated and buried beneath the pectoralis major to prevent the proximal stump from involvement in the reinnervation process. In group III, the transected median nerve was not anastomosed. The wound was closed with 4-0 nylon skin sutures.

Functional recovery was measured using the grasping test and the finger flexor muscle contraction force test. Morphological changes were assessed by comparing the number of regenerated myelinated axons.

#### Grasping test

**Fig. 1:**
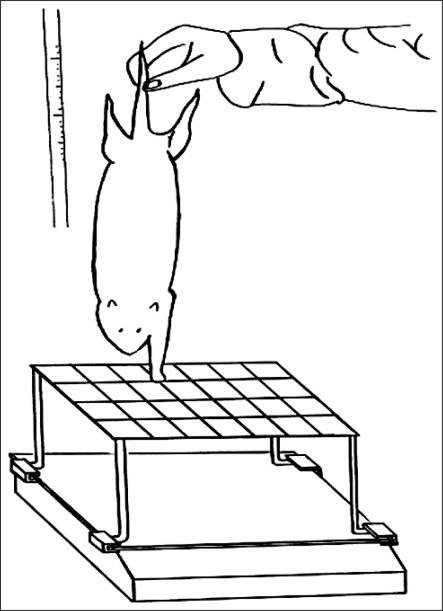
A rat underwent a grasping strength test. The animal was gently lifted by the tail and allowed to grasp the grid connected to an electronic balance. While grasping, the rat continued to be lifted by the tail with increasing firmness until it loosened its grip. At that precise moment, the negative value demonstrated by the balance was recorded.

The grasping strength was measured10) preoperatively, 10 and 20 weeks after the surgical procedure. Animals were gently lifted by the tail and allowed to grasp the grid of a cage with their forepaws. Finger flexion then was observed. For the assessment of the grasping strength, a 15 × 15 cm square wire grid, 1.5 mm in diameter, was connected to an electronic balance [pull and push gauge (model AK-1, Algol, Nagano, Japan)] using a clamp and rod designed for the grid. The rats were allowed to grip the grid, while being pulled vertically upward by the tail at a constant rate of 10 mm/s, until the grip loosened. At the precise moment of release, the maximal negative value registered on the gauge was recorded (Fig. 1). Each rat was tested three times and the average value was documented. When the grid was raised by the wrist or the nail, it was ignored. At the 10th post-surgical week, each 2 cm segment of the median and ulnar nerve, in the contralateral forepaws, was resected to prevent gripping the grid^9^-11) and to measure the number of myelinated axons in the normal nerve. At 20 weeks postoperatively, after the grasping test, the nerve coapted site was exposed and the muscle contraction force test and a nerve biopsy were performed under anesthesia.

#### Contraction strength test for finger flexor muscle

For the assessment of the finger flexor muscle contraction strength, the volar aspect of the forearm was incised and then the tendon and muscle belly of the flexor were exposed. The finger flexor tendon was connected to a tension transducer by 3-0 non-absorbable sutures. The forearm and wrist were fixated with an 18 gauge needle. The electrical stimuli (60 Hz, 1 ms in duration and 25 V) from a Harvard stimulator (Harvard Apparatus Ltd, Edenbridge, UK) were delivered via bipolar electrodes to the mid-belly of finger flexor muscle. The isometric muscle contraction strength was recorded using a Harvard tension transducer and was displayed on an oscilloscope.^6^,^12^,^13^)

#### Morphologic evaluation

**Fig. 2:**
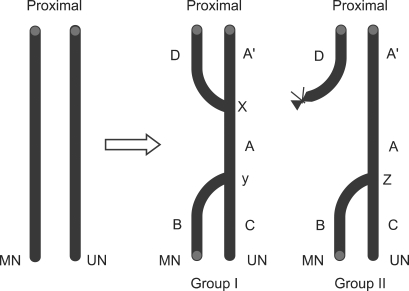
Schematic drawing of the end-to-side neurorrhaphy in Group I and Group II. MN: Median nerve, UN: Ulnar nerve.

After completing the measurement of force, a nerve biopsy was performed at A, A', B, C and D in group I (double ETS neurorrhaphy) while at A, B and C in group II (ETS neurorrhaphy)(Fig. 2). In group I, if the proximal coaptation point was x, the distal neurorrhaphy site was y. A was between × and y in the ulnar nerve, B was distal to y in the median nerve, C was distal to y in the ulnar nerve, D was proximal to x in the median nerve, and A' was proximal to x in the ulnar nerve. In group II, when the coaptation site was z, A was proximal to z in the ulnar nerve, B was distal to z in the median nerve, and C was distal to z in the ulnar nerve. Since the proximal stump of the median nerve was not coapted to the proximal ulnar nerve in group II, the site corresponding to A' in group I was similar to A in group II. Therefore, we ignored the test at A' in group II.

The nerve tissues were prepared for optical microscopy.^7^,^8^,^12^,14) Axonal counts were performed by a single laboratory technician, blinded to the specimen identity.

#### Data analysis

All data were analyzed using the SPSS ver. 11.0 (SPSS Inc., Chicago, IL, USA). The Kruskal-Wallis test was used for the differences in grasping strength and flexor muscle contraction force and compared between the two groups. The differences in the number of myelinated axons were evaluated using the Wilcoxon signed-rank test. The power of the test was 81% with a 0.05 significance level.

## RESULTS

### Grasping Test

The normal grasping strength in the forepaw before surgery was 581 ± 103 g. In group I (double ETS neurorrhaphy), the average was 137 ± 30 g at 10 weeks postoperatively and 155 ± 40 g at 20 weeks postoperatively. In group II (single ETS neurorrhaphy), it was 45 ± 19 g and 97 ± 31 g, respectively (Table 1). In group III, it was 0 g at both testing times. The differences between the groups were statistically significant both at postoperative week 10 (*p* = 0.001) and 20 (*p* = 0.002).

### Finger Flexor Muscle Contraction Strength

At postoperative week 20, the muscle contraction force was 45.4 ± 5.6 g in group I and 30.2 ± 5.6 g in group II (Table 1). The differences between the groups were statistically significant (*p* = 0.004).

### The Number of Myelinated Axons

**Fig. 3:**
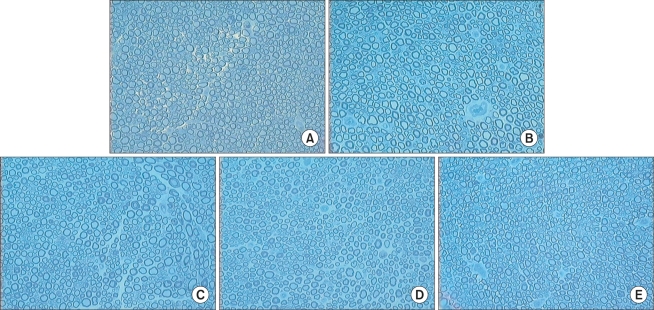
Photomicrographs of the cross-section in Group I. (A) Site A. Average number of the myelinated nerve fibers was 1760 ± 310. (B) Site B. Average number of the myelinated nerve fibers was 1205 ± 233. Regenerated nerve fibers were more compact with increased axonal size than in Group II-site B. (C) Site C. Average number of the myelinated nerve fibers was 1713 ± 341. (D) Site D. Average number of the myelinated nerve fibers was 1643 ± 215. (E) Site A'. Average number of the myelinated nerve fibers was 1958 ± 209 (Toluidine blue, × 200).

**Fig. 4:**
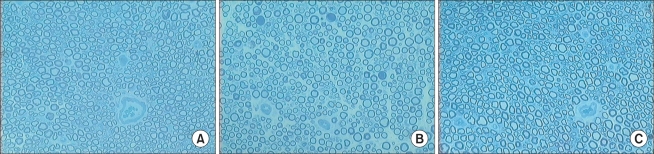
Photomicrographs of the cross-section in Group II. (A) Site A. Average number of the myelinated nerve fibers was 1895 ± 213. (B) Site B. Average number of the myelinated nerve fibers was 536 ± 184. (C) Site C. Average number of the myelinated nerve fibers was 1800 ± 264 (Toluidine blue, × 200).

The number of myelinated axons in the median and ulnar nerve of a normal rat was 2283 ± 223 and 1495 ± 268 respectively. In group I, the number of myelinated nerve fibers was 1760 ± 310 at A (Fig. 3A), 1205 ± 233 at B (Fig. 3B), 1713 ± 341 at C (Fig. 3C), 1643 ± 215 at D (Fig. 3D), and 1958 ± 209 at A' (Fig. 3E). In group II, it was 1895 ± 213 at A (Fig. 4A), 536 ± 184 at B (Fig. 4B), and 1800 ± 264 at C (Fig. 4C, Table 2).

No statistical significance was observed in the number of axons between the two groups at A (*p* = 0.175) and at C (*p* = 0.441), but the number of axons was significant in comparisons with B (*p* = 0.002). The difference in the number of axons at site A and C was not statistically significant for each group.

## DISCUSSION

It is widely accepted that spontaneous regeneration of nerves occurs relatively often in small animals such as the rat. Therefore, care was taken to prevent the proximal stump of the transected median nerve from reattaching spontaneously to the ulnar nerve, or to the distal portion of the injured median nerve. Such reconnection was not observed in groups II and III.

As in humans, anatomical variations between the median and ulnar nerve in rats must be taken into consideration. While Martin-Gruber anastomosis, an anomalous median-to-ulnar nerve communication in the forearm, is known to be present in 17% of the population,15) its prevalence in rats has not been studied. Bertelli et al.9) reported that transection of the ulnar nerve would be a safe procedure in studies involving the median nerve in a rat model. In this study, there was no choice but to preserve the ulnar nerve as it was used as the donor nerve for the ETS neurorrhaphy. Since the grasping strength was 0 g in group III, the above mentioned anomaly was thought to not interfere with our results. The measurement of grasping strength had the technical problem of flexion of the wrist being affected by the flexor carpi ulnaris innervated by the ulnar nerve; this could have interfered with accurate measurement of the grasping strength of the fingers. Therefore, the grid space was made narrow enough to prevent the wrist from being trapped in the wire.

The ETS neurorrhaphy offers two theoretical benefits. First, it does not sacrifice the donor nerve that replaces a nerve defect. Second, suturing the distal stump of the injured nerve to a healthy donor nerve, in an end-to-side fashion, promotes recovery and prevents motor end-plate atrophy that could occur during the reinnervation period through a nerve graft for end-to-end neurorrhaphy in cases with a long segmental defect.

In recent studies regarding ETS neurorrhaphy, concerns about the analysis of factors and variables that enhance axonal sprouting after the procedure have been raised; these include the cross section size of the epineurial window, the number of Schwann cells at the coaptation site, the angle between the donor nerve and the recipient nerve, the helicoid technique, timing of coaptation, the growth factors at the coaptation site, and the type of reinnervated muscle.^5^,^16^,17) Indeed, promoting axonal sprouting directly correlates with reinnervation through the distal stump in the ETS neurorrhaphy. Therefore, increasing axonal sprouting is the most important factor for the clinical outcome of this technique.^5^-^8^,^13^,^14^,16)

The reinnervation mechanism after ETS neurorrhaphy has not been clearly identified. Experiments on the origin of new formed axons have yielded different results. For convenience, the current theories can be broadly categorized into three explanations.^8^,^18^-23)

First, the axons regenerated from the original neurons can reconnect to the original target organ by chance. This can be assumed especially when a nerve stump is anastomosed to the trunk of the spinal cord at a similar level. This theory is supported by experiments that showed that regeneration cannot occur if the recipient and the donor nerve originate from different spinal cord levels,21) and the number of myelinated axons increased in the donor nerve at the proximal site of the ETS neurorrhaphy.22) In the present study, the number of myelinated axons at the proximal site of the ETS coaptation of the ulnar nerve was 1760 in group I and 1895 in group II, while it was 1495 in the normal rats. These findings suggest that the proximal stump of the severed nerve contributed to the axonal regeneration.

Second, collateral axonal sprouting from a donor nerve can connect with a recipient nerve.^8^,^19^,23) According to some investigators, a donor nerve appears to cut offits nerve branch connected to the original target organ in order to create a functional connection to a recipient nerve.12) This idea is based on experiments showing the downgrading of the donor nerve distal to the coaptation site with no functional connection between the reinnervated muscle and the one innervated by the donor nerve. The results of the present study indicated that collateral axonal sprouting played an essential role in nerve repair. There was no significant difference in the number of myelinated axons in the ulnar nerve (donor nerve) at site A of each group, but the number of myelinated axons at site B in group I was almost two times the number in group II, 1205 and 536 respectively. However, downgrading of the donor nerve distal to the coaptation site was not observed.

Third, terminal sprouting from the injured donor nerve during the construction of an epineurial window or performing the ETS neurorrhaphy can reinnervate the target organ. However, this theory is not widely accepted because degenerative changes of the donor nerve distal to the coaptation site occur rarely. The difference in the number of myelinated axons between the proximal and distal sites in relation to the ETS neurorrhaphy donor nerve was not statistically significant in the present study.

Various models designed to prevent the proximal stump from interfering with the evaluation of the efficacy of the ETS neurorrhaphy have been suggested. However, no prior studies have investigated whether the proximal stump can facilitate the regeneration process. Prior studies have focused on the findings that support regeneration after an ETS neurorrhaphy. By contrast, we sought to use the proximal stump to determine whether it facilitated the nerve repair process after an ETS neurorrhaphy in cases with long segmental nerve defects where the proximal stump is available.

We performed experiments to assess the efficacy of a double ETS neurorrhaphy in a rat model. With regard to the grasping force, the flexor muscle contraction strength and the axonal regeneration, our findings showed that the method used was more effective for peripheral nerve reconstruction than the conventional ETS neurorrhaphy. These results imply that the contribution from the proximal nerve stump in the double end-to-side nerve repair facilitated axonal sprouting from the donor nerve and aided in improved functional recovery in an end-to-side coaptation model.

## Figures and Tables

**Table 1:**
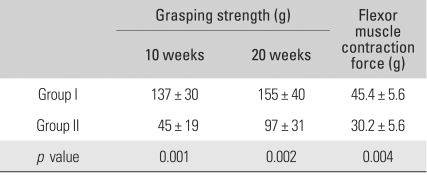
Comparison of Grasping Strengths and Flexor Muscle Contraction Forces in Group I and II

**Table 2:**
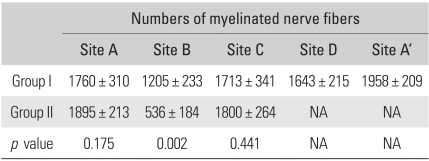
Comparison of Numbers of Myelinated Nerve Fibers in Group I and II

NA: Not available.
